# Molecular, Pathophysiological, and Clinical Aspects of Corticosteroid-Induced Neuropsychiatric Effects: From Bench to Bedside

**DOI:** 10.3390/biomedicines12092131

**Published:** 2024-09-19

**Authors:** Sara Sofía-Avendaño-Lopez, Angela Johanna Rodríguez-Marín, Mateo Lara-Castillo, Juanita Agresott-Carrillo, Luna Estefanía Lara-Cortés, Juan Felipe Sánchez-Almanzar, Sophya Villamil-Cruz, Luis Carlos Rojas-Rodríguez, Daniel Felipe Ariza-Salamanca, Mariana Gaviria-Carrillo, Carlos Alberto Calderon-Ospina, Jesús Rodríguez-Quintana

**Affiliations:** 1 Social Epidemiology Research Team, Institut Pierre Louis d’Epidémiologie et de Santé Publique, INSERM, Sorbonne Université, F 75012 Paris, France; 2 Pharmacology Unit, Department of Biomedical Sciences, School of Medicine and Health Sciences, Universidad del Rosario, Bogotá 111221, Colombiacarlos.calderon@urosario.edu.co (C.A.C.-O.); 3 Center for Research in Genetics and Genomics (CIGGUR), Institute of Translational Medicine (IMT), School of Medicine and Health Sciences, Universidad del Rosario, Bogotá 111221, Colombia; 4 Research Group in Applied Biomedical Sciences (UR Biomed), School of Medicine and Health Sciences, Universidad del Rosario, Bogotá 111221, Colombia; 5 Department of Pharmacobiology, Center for Research and Advanced Studies (Cinvestav), National Polytechnic Institute, Mexico City 14300, Mexico; daniel.ariza@cinvestav.mx; 6 Neuroscience Research Group (NeURos), NeuroVitae Center for Neuroscience, School of Medicine and Health Sciences, Universidad del Rosario, Bogotá 111221, Colombia; mariana.gaviria@urosario.edu.co; 7 Fundacion CardioInfantil-Instituto de Cardiología, Bogotá 111156, Colombia; 8 Hospital Universitario Mayor Mederi, Bogotá 111411, Colombia

**Keywords:** glucocorticoids, neuropsychiatry, adverse effects, anxiety, depression, psychotic disorders, pharmacovigilance, receptors, GABA-A, receptors, N-methyl-D-aspartate

## Abstract

Corticosteroids are frequently prescribed across medical disciplines, yet they are associated with various adverse effects, including neuropsychiatric symptoms, documented since their introduction over 60 years ago. The cellular mechanisms underlying neuropsychiatric symptoms are complex and somewhat obscure, involving multiple pathways. Notably, they include changes in excitability, cellular death of hippocampal and striatal neurons, and increased inflammation and oxidative stress. Clinical presentation varies, encompassing affective disorders (anxiety, euphoria, depression), psychotic episodes, and cognitive deficits. It is crucial to note that these manifestations often go unnoticed by treating physicians, leading to delayed detection of severe symptoms, complications, and underreporting. Discontinuation of corticosteroids constitutes the cornerstone of treatment, resolving symptoms in up to 80% of cases. Although the literature on this topic is scant, isolated cases and limited studies have explored the efficacy of psychotropic medications for symptomatic control and prophylaxis. Pharmacological intervention may be warranted in situations where corticosteroid reduction or withdrawal is not feasible or beneficial for the patient.

## 1. Introduction

Corticosteroids are integral to treatment across various medical domains. They are synthetic analogs of natural steroid hormones produced by the adrenal cortex like glucocorticoids (GCs), mineralocorticoids and sexual hormones (testosterone and androstenolone) [1]. The mechanism of action of corticosteroids involves the activation of intracellular glucocorticoid receptors, which act as ligand-activated transcription factors, thereby modifying gene transcription in various ways [2,3]. These alterations in gene expression, along with other non-genomic effects, confer upon GCs a wide range of clinical applications, particularly in managing inflammatory and autoimmune conditions, adrenal insufficiency, and certain malignancies [1,2,3,4]. These drugs, in low doses, can also be useful in the treatment of adrenal insufficiency. However, it should not be forgotten that if patients use corticosteroids (GC) chronically and are abruptly discontinued, they could also develop adrenal insufficiency [5].

Despite their significant clinical utility, adverse reactions are common, occurring in up to 90% of patients with prolonged use (over 60 days) [5]. The widespread distribution of glucocorticoid and mineralocorticoid receptors throughout the body means that adverse reactions can affect multiple systems, including the central nervous system [6,7]. Excessive glucocorticoid use can overwhelm intrinsic mineralocorticoid receptor mechanisms, contributing to the development of adverse events in systems where these receptors play a regulatory role [5]. Corticosteroid-induced psychiatric disorders (CIPDs), although well documented since the inception of corticosteroid therapy, often remain challenging to detect and frequently go unnoticed [1,7].

Historically, the association between corticosteroids and the development of neuropsychiatric symptoms has been termed “steroid psychosis”, with initial presentations often including acute delirium and dysphoria [8]. However, these manifestations extend beyond psychotic episodes to include mania, depression, delirium, and an increased risk of suicide [9]. In 1983, Lewis and Smith reported that depression is the most common psychiatric alteration, with only 14% of cases presenting isolated psychosis [10].

The primary strategy for managing these psychiatric disorders is the reduction or discontinuation of corticosteroids [10]. However, in some clinical scenarios, this approach is not feasible, necessitating the use of psychotropic medications for symptomatic control [10]. Since the initial documentation of corticosteroid-induced psychiatric disorders, various medications have been used, although the literature primarily contains case series and limited controlled studies demonstrating their efficacy [11].

In this review, we aim to delve into the key pathophysiological aspects of CIPDs, identify the most frequently implicated medications, and explore any relationships with dosage or duration of administration. We will also examine risk factors for developing these adverse reactions and discuss therapeutic strategies described in the literature for managing this condition.

## 2. Cellular and Molecular Mechanisms Leading to Neuropsychiatric Symptoms Induced by Corticosteroids

**Figure 1:**
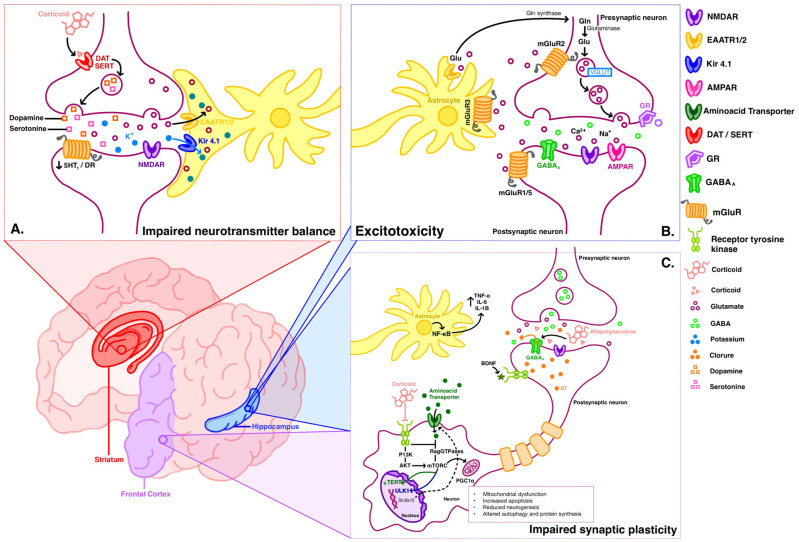
Cellular and molecular mechanisms leading to neuropsychiatric symptoms induced by corticosteroids. Cellular and molecular mechanisms leading to neuropsychiatric symptoms can be divided either by anatomical region or by ongoing subcellular pathological processes. (**A**) Impaired neurotransmitter balance exemplifies how corticosterone reduces synaptic availability of endogenous amines and catecholamines in the striatum. This also considers the tripartite synapse and how the increased release of glutamate and K^+^ disrupts the homeostatic role of astrocytes. (**B**) Excitotoxicity in the hippocampus. Excitotoxicity occurs when increased calcium influx through NMDA receptors leads to cell death, and it also involves prior alterations in glycine-glutamine-glutamate metabolism. The hippocampus is particularly susceptible to corticosteroids, displaying a U-shaped concentration-response curve. (**C**) Impaired synaptic plasticity in the hippocampus and frontal cortex. Various mechanisms contribute to reduced neurogenesis, proliferation, differentiation, and survival. The illustration highlights increased activation of the mTOR pathway, decreased function of amino acid transporters, increased intracellular chloride, and heightened inflammation. Taken together, these mechanisms partially explain the emergence of complex symptoms such as depression, anxiety, psychosis, and cognitive impairment. Abbreviations: 5HT_1_: 5-hydroxytryptamine receptor 1, AKT: Protein Kinase B, AMPAR: α-amino-3-hydroxy-5-methyl-4-isoxazolepropionic acid receptor, BDNF: Brain-Derived Neurotrophic Factor, DAT: Dopamine Transporter, DR: Dopamine Receptor, EAATR: Excitatory Amino Acid Transporter, GABA_A_: Gamma-Aminobutyric Acid Type A receptor, GR: Glucocorticoid Receptor, IL: Interleukin, Kir: Inwardly Rectifying Potassium Channels, mGluR: Metabotropic Glutamate Receptor, NF-kB: Nuclear Factor kappa B, NMDAR: N-methyl-D-aspartate receptor, PI3K: Phosphoinositide 3-kinase, PGC1a: Peroxisome Proliferator-Activated Receptor Gamma Coactivator 1-alpha, SERT: Serotonin Transporter, TERT: Telomerase Reverse Transcriptase, TNF-α: Tumor Necrosis Factor-alpha, ULK1: Unc-51 Like Autophagy Activating Kinase 1, VGLUT: Vesicular Glutamate Transporter.

This section reviews the literature on changes in neurotransmission, neurogenesis, and neuronal differentiation within the hippocampus, cortex, and corticostriatal connections following corticosteroid exposure and stressful environments. Readers are encouraged to familiarize themselves with the detailed physiological functions of these structures and circuits beforehand, as this section focuses on the pathological mechanisms. Figure 1 shows the Cellular and molecular mechanisms leading to neuropsychiatric symptoms induced by corticosteroids.

### 2.1. Neurotransmission and Neurogenesis

Physiologically, corticosteroids cross cell membranes freely and activate intracellular receptors responsible for initiating the transcription of genes related to inflammation and metabolism. In the central nervous system (CNS), corticosteroids can activate tyrosine hydroxylase [12], monoamine oxidase [13], and dopamine β-hydroxylase [14], all of which are involved in the production and metabolism of endogenous amines. In essence, corticosteroids regulate the expression of enzymes and hormones that modulate neurotransmitter and hormonal systems. Consequently, exogenous corticosteroid administration can alter cognition and behavior [15].

There are multiple preclinical models of depression, and in this context, sub-chronic administration of corticosteroids is often employed. Various protocols, with subtle differences, are used to induce depression in animal models, with exposure durations ranging from 7 days to 7 weeks and doses up to 40 mg/kg of corticosterone [16,17]. Chronic daily administration of corticosterone has been shown to affect multiple signaling pathways, gene expression, and protein synthesis related to depressive behavior. This behavior is typically assessed using the forced swim test (FST), tail suspension test (TST), and evaluation of coat state deterioration. The FST and TST are widely used to assess depressive-like behavior in rodents by measuring immobility, which reflects behavioral despair. In the FST, rodents are placed in water, and in the TST, they are suspended by the tail, with immobility indicating a depressive state. The evaluation of coat state deterioration involves assessing the animal’s fur for signs of neglect, often linked to stress or poor general well-being, commonly used to gauge depression or illness effects. Key findings indicate that corticosterone treatment leads to decreased levels of serotonin and norepinephrine, reduced neurogenesis, increased inflammatory response, and impaired buffering capacity for reactive species [18,19].

On the other hand, chronic corticosteroid use has been associated with atrophy of the amygdala, a critical brain region involved in emotion regulation and autonomic reflexes. This atrophy may impair the ability to manage stress and emotions and disrupt autonomic responses, such as the “fight or flight” reaction. The amygdala’s role in fear response and autonomic nervous system modulation suggests that its atrophy could have significant implications for the emotional and physical well-being of patients [20].

### 2.2. Effects on the Hippocampus

Research has shown that hippocampal function is sensitive to glucocorticoids, with fluctuations in their levels significantly affecting cognitive processes [21]. Depression is characterized by significant alterations in hippocampal structure, with a reduction in volume correlating with disease severity in patients [16,22]. Although the exact mechanisms are not fully understood, a U-shaped concentration-response curve has been proposed to explain this relationship. Sustained corticosterone administration has numerous effects on the hippocampus as well [23]. Reduction in hippocampal volume may result from increased cell death, likely due to alterations in mTOR signaling and heightened oxidative stress. Olescowicz et al. [24] utilized a female mouse model with chronic administration of 20 mg/kg of corticosterone over 21 days, observing decreased overall hippocampal volume and increased phosphorylation of downstream effectors in the mTOR pathway. Dysregulation of mTOR signaling can induce cell death through inhibition of autophagy [25], oxidative stress leading to mitochondrial dysfunction [26], and disruption of cell homeostasis leading to programmed cell death [27].

Interestingly, the reduction in hippocampal volume was prevented when animals were pretreated with agmatine (0.1 mg/kg p.o.) and fluoxetine (10 mg/kg p.o.) for 21 days [24]. Agmatine, a natural metabolite of arginine, is known for its modulation of cardiovascular function and metabolic processes. The exact mechanisms by which agmatine reduces cell death are not fully elucidated; however, it has been shown to activate signaling cascades through PI3K/AKT and MEK/ERK pathways, which are implicated in cellular survival, proliferation, synaptic plasticity, and differentiation [28,29]. Fluoxetine shows similar effects to agmatine, suggesting that this selective inhibitor sertraline might also be having non-synaptic effects. The effects of fluoxetine in a similar preclinical model were assessed by David et al. [30]. They found that fluoxetine enhances neurogenesis, including proliferation, differentiation, and survival. Additionally, fluoxetine exhibits antidepressant effects independent of neurogenesis. The researchers observed a decrease in mRNA levels of β-arrestin 1 and 2, as well as Giα2, following chronic administration of corticosterone; these effects were reversed by chronic fluoxetine treatment. These mRNAs are involved in GPCR signaling, particularly in relation to the CRF1 receptor.

Liu et al. [31], employing a model of chronic corticosterone administration (10 mg/kg for 28 days) in adult male C57BL/6Ja mice, found that varying concentrations of glucocorticoids modulate the development of neural stem cells and cell growth in the dentate gyrus of the hippocampus bidirectionally. The negative modulation of neurogenesis was observed to be dependent on telomerase activity, as evidenced by the downregulation of TERT activity following exposure to high concentrations (10 μM) of corticosterone. Moreover, the administration of metyrapone (100 mg/kg, i.p.), a glucocorticoid synthesis blocker, restored TERT activity and alleviated depressive symptoms in mice. Neurogenesis has consistently been shown to be impaired in corticosterone-induced depression [32].

Chronic corticosteroid administration disrupts neurotransmitter balance. Herbet et al. demonstrated increased oxidative DNA damage in isolated prefrontal cortices of mice receiving daily corticosterone, attributed to reduced mRNA expression of *Fkbp5*, *COMT*, *Adora1*, and *Slc6a15* [33]. These genes are crucial for cellular metabolism, monoamine breakdown, glycine reuptake [34], and other processes impacting neurotransmitter balance. Co-administration of corticosterone with edaravone (10 mg/kg) or paroxetine (10 mg/kg) increased *Slc6a15* mRNA expression and improved behavioral tests, although it did not affect DNA oxidative damage. This suggests that behavioral improvements may relate to improved neurotransmitter reuptake but not directly to overall cellular process improvements [33].

Several preclinical studies have demonstrated that enhancing antioxidant mechanisms can alleviate depression. Chronic corticosterone administration impairs behavioral performance, reduces BDNF levels, increases oxidative stress, and diminishes antioxidant activity in the mouse hippocampus [35]. Administration of RIP-2 has shown promise in improving behavioral outcomes and reducing oxidative damage. Riparins, benzamide alkaloids from *Aniba riparia* (Nees), are reported to possess anxiolytic, antidepressant, and antioxidant properties [35,36].

### 2.3. Inflammation and Oxidative Stress

Increased inflammation is another key feature of corticosterone-induced depression. Ge et al. [37] evidenced an increase in inflammatory cytokines IL-1β, IL-6, and TNF-α in the isolated hippocampus and prefrontal cortices of C57BL/6J male mice subjected to subcutaneous treatment with 20 mg/kg of corticosterone. Interestingly, cotreatment with quercetin, a known flavonoid with antioxidant and anti-inflammatory properties, reduced proinflammatory cytokines in these depressed animals. Similarly, in a lipopolysaccharide-induced depression model, quercetin also reduced inflammation and caspase-3 activation in the hippocampus [38].

Impaired synaptic plasticity is another hallmark of corticosterone-induced depression and cognitive impairment [39,40]. Recent evidence has demonstrated that perineuronal nets around fast-spiking GABAergic neurons play a role in mood regulation [41]. The CA3 subregion of the hippocampus is key for the contextualization of episodic memories. Parvalbumin-positive (PV) basket cells inhibitory neurons are sensitive to changes in spatial enriched environment versus fear conditioning restricted space.

When animals are in an enriched environment PV neurons are relatively low when compared to a more adverse space. The activation of PV cells promotes a high-PV state and impedes hippocampal plasticity [40]. Furthermore, perineuronal nets also enhance a high PV state. These perineuronal nets are increased by corticosterone treatment [42]. Coutens et al. [40] evidenced a reduction in parvalbumin-positive neurons surrounded by perineuronal nets when corticosterone was co-administered with venlafaxine chronically, meaning venlafaxine resets hippocampal plasticity by decreasing the high PV state. Interneurons play a critical role in long-term potentiation (LTP) and long-term depression (LTD), which are key mechanisms of synaptic plasticity. Interneurons also modulate synchrony and overall circuit excitability. The interaction between brain regions generates complex cognitive phenomena such as consciousness and emotions, which are impaired in patients with depression.

Brain-Derived Neurotrophic Factor (BDNF) expression is essential for neuronal plasticity, as it modulates synaptic plasticity by promoting synapse formation and strengthening, enhancing LTP, and supporting dendritic growth and branching, all of which are critical for learning, memory, and cognitive performance [43]. Lin et al. [39] observed differential changes in proBDNF and mature BDNF levels in various brain regions following chronic corticosterone administration. Specifically, proBDNF levels increased in the hippocampus and cerebellum and were correlated with depressive and anxiety-related behaviors, as measured by the splash test and open field test. The splash test is used to evaluate grooming behavior in rodents, typically as an indicator of self-care or motivational deficits, often associated with depressive-like states. The open field test assesses general locomotor activity and exploratory behavior, as well as anxiety-related responses, such as thigmotaxis (the tendency to remain close to the walls of the arena), which reflects a natural aversion to open spaces. In the hypothalamus, proBDNF levels were negatively correlated with sucrose consumption, indicating a potential link between BDNF dysregulation and anhedonia. These findings underscore the importance of BDNF in maintaining normal hippocampal function and effective synaptic plasticity.

Interestingly, in a similar depression model, a low dose of thymol (50 mg/kg p.o.)—a monoterpenoid phenol—was shown to improve performance in the FST, TST, sucrose preference test, and other behavioral tests related to anxiety-like and depressive-like behaviors. Additionally, thymol restored BDNF levels in the hippocampus of female mice in a similar fashion as fluvoxamine, an SSRI [44]. Similarly, Sawamoto et al. [45] found that 3,5,6,7,8,3′,4′-heptamethoxyflavone, a flavonoid, restores BDNF levels, neurogenesis, and neuroplasticity in the hippocampus of a corticosterone-treated mouse model. This study also demonstrated the role of ERK activation in synaptic plasticity. From a translational perspective, pharmacological agents with anti-inflammatory and antioxidant properties such as thymol and 3,5,6,7,8,3′,4′-heptamethoxyflavone might arise as a complementary therapeutic approach to patients with depression. Nonetheless, much is still to be considered in terms of drug dose, pharmacological interactions and therapeutic index of these compounds.

### 2.4. Mechanisms of Mania and Psychosis

Mania is a common neuropsychiatric symptom often induced by the exogenous and acute administration of corticosteroids [46,47]. However, the mechanisms leading to this symptom remain poorly understood. Valvassori et al. [48] employed a paradoxical sleep deprivation protocol known to induce hyperactivity in mice, resembling mania-like behavior [49]. They observed increased levels of corticosterone and adrenocorticotropic hormone, lipid peroxidation, DNA oxidative damage, alterations in antioxidant enzymes, and heightened inflammation in the hippocampus and prefrontal cortex of male C57 mice after 5 days of sleep deprivation. Concurrent treatment with lithium (47.3 mg/kg daily) reduced inflammation and oxidative stress, as well as diminished locomotor hyperactivity. Lithium has consistently been shown to modulate antioxidant enzymes and reduce inflammation [50,51,52] and has been widely used in clinical practice for a long time.

Neuroactive steroids (NS) such as allopregnanolone, pregnenolone, and dehydroepiandrosterone modulate GABAergic and glutamatergic transmission, neuroinflammation, and neuroplasticity. Various alterations in NS levels have been observed in individuals with unipolar depression and bipolar disorder. Increased levels of pregnenolone and dehydroepiandrosterone have been reported in the posterior cingulate and parietal cortex of patients with bipolar disorder [53]. NS has also demonstrated neuroprotective effects, specifically protecting against apoptosis induced by Bcl-2. This protein regulates apoptosis by inhibiting cell death and promoting cell survival [54]. Marx et al. [55] showed that chronic lithium treatment enhances pregnenolone levels in the prefrontal cortex of Bcl-2 knockout mice compared to those treated with a vehicle. Additionally, lithium treatment has been shown to increase neurogenesis in the rat hippocampus by elevating Bcl-2 levels and enhancing neuronal differentiation [56].

Symptoms resembling mania, such as hyperlocomotion, sleep disturbances, and anxiety-like behavior, have been observed in a preclinical mouse model heterozygous for cathepsin D deficiency. This enzyme is crucial for protein degradation [57]. Interestingly, under stressful conditions, these mice can exhibit anhedonia, helplessness, and depression-like behavior, along with increased levels of corticosterone. Cathepsin D dysfunction has been moderately linked to neurodegeneration and may contribute to the development of neuropsychiatric symptoms [58,59]. However, the relationship between cathepsin D function and corticosterone levels is not fully understood. It may be related to the U-shaped concentration-response curve mentioned earlier.

Psychosis is defined as a collection of psychological symptoms that result in a loss of contact with reality and is a common symptom following acute corticosteroid exposure [15,60]. Dysfunction in dopamine circuits is traditionally considered a key substrate of psychosis. Dopaminergic pathways involve anatomical structures such as the striatum, brainstem, and frontal cortex. In experimental settings, it has been shown that corticosteroids induce apoptosis in striatal cells and pericytes [61,62]. Mitchell et al., using male Sprague Dawley rats, demonstrated that a single administration of a high dose (20 mg/kg) of dexamethasone intraperitoneally significantly reduced the number of striatal neurons in the dorsomedial striatum, with striatopallidal neurons being particularly susceptible to apoptosis. The authors suggested that this effect may be related to the fact that most of the inputs to the dorsomedial striatum come from the cingulate cortex, which regulates stress-induced glucocorticoid release [61,63]. Katychev et al. [62], showed that a high concentration (2 × 10^−3^ M) of dexamethasone in cultivated microvascular pericytes induces significant apoptosis. It is important to consider that in these experimental conditions, dexamethasone is administered in doses that do not resemble typical clinical use. However, these experimental findings highlight the significant role of glucocorticoids in cell survival.

Not only can acute corticosteroid exposure induce psychosis, but early life stress has also been shown to cause lasting damage to brain regions involved in dopaminergic pathways, increasing the risk of developing psychotic disorders. In a study, rats separated from their mothers between postnatal days 2 and 14 to induce stress were used to examine the expression of stress-related factors in the substantia nigra, ventral tegmental area, and dorsal and ventral striatum [64]. The researchers found altered mRNA expression of stress-related factors (FKBP5 and PTGES3) in the ventral tegmental area. Specifically, FKBP5 levels were decreased, while PTGES3 levels were increased in stressed rats. The authors proposed that these changes could enhance neuronal responsiveness to cortisol by promoting the binding of cortisol to high-affinity glucocorticoid heterocomplexes and/or facilitating the translocation of glucocorticoid receptors into the nucleus. Additionally, mRNA levels of BDNF I and BDNF IIC were increased in female rats, which were significantly less affected than male rats, highlighting the role of BDNF in neuroprotection.

Glucocorticoids modulate the stress-induced epigenetic regulation of dopaminergic neurons. When environmental stressors combine with genetic mutations, the result can be complex disorders such as schizophrenia. Niwa et al. [65] demonstrated that a combination of “suboptimal” exposure to an environmental stressor and specific genetic risks can lead to significant social interaction impairments. Their study showed that a 3-week isolation period after birth in a transgenic mouse model with a dominant-negative DISC1 (disrupted in schizophrenia 1) under the control of the prion protein promoter (DISC1-DN-Tg-PrP) led to severe impairments on the prepulse inhibition test, increased immobility time in the FST, and altered locomotor activity in DISC1-DN-Tg-PrP adolescent mice. Additionally, a decrease in tyrosine hydroxylase expression and an increase in D2R expression were observed in the frontal cortex of these animals.

Neuroactive steroids (NS) also play a role in regulating cognitive acuity. Busquets-Garcia et al. found that in a model of cannabis-induced acute psychotic-like states, administration of pregnenolone (6 mg/kg, s.c.) reduced cognitive impairment, social interaction deficits, and hyperlocomotion, which are often used in preclinical research to approximate positive symptoms of drug-induced psychotic-like states [66]. Notably, the authors showed that the effects of pregnenolone on psychotic-like states are mediated by inhibition of the CB1 receptor. Overall, the evidence indicates that brain regions associated with dopaminergic pathways are highly sensitive to corticosteroid actions during crucial periods of central nervous system development and maturation. This underscores the role of corticosteroids as key regulators of optimal brain maturation and both motor and cognitive performance.

### 2.5. Excitotoxicity

Excitotoxicity is a pathological process in which excessive stimulation of glutamate receptors results in neuronal injury and death. This overactivation leads to a harmful influx of calcium ions, triggering intracellular events that culminate in cell damage. Excitotoxicity has been implicated as a mechanism of cell damage following corticosteroid administration and in chronic stress [67]. Both mineralocorticoid and glucocorticoid receptors, which can be expressed in the cytoplasm and membrane of neurons, mediate genomic and non-genomic effects on synaptic transmission [68].

Karst et al. [69], using in vitro preparations of CA1 hippocampal neurons and patch-clamp recordings, demonstrated that 100 nM extracellular corticosterone quickly and transiently enhances the frequency of miniature excitatory postsynaptic potentials (mEPSPs) without affecting their amplitude or kinetics. These experiments, conducted with antagonists of classical receptors implicated in EPSPs and in animals with knockout MR and GR, indicate that corticosterone modulates neurotransmitter release probability via glutamate transmission, likely involving metabotropic glutamate receptors (mGluRs) rather than NMDA receptors. Similar findings were observed in prefrontal cortex preparations [70,71]. Chronic stress has been shown to impair neuronal and glial function, potentially leading to excitotoxicity through disrupted glutamate uptake by excitatory amino acid transporters and altered glycine-glutamine-glutamate conversion [72]. Collectively, these findings illustrate how corticosteroids modulate glutamate transmission, with cellular death arising from increased glutamate concentration in the synaptic cleft [73].

This section reviewed the available literature regarding the main mechanisms implicated in the genesis of the most common CIPDs. Preclinical evidence consistently shows that the main anatomical areas affected by glucocorticoids are the hippocampus, the prefrontal cortex, and the striatopallidal neurons. These areas are clearly related to emotional control and cognitive processing. Regardless of whether the exposure to corticosteroids is acute or chronic, cellular death is caused by an override of homeostatic control mechanisms related to redox state, inflammation, and calcium-related signaling. The adverse effects arising after corticosteroid exposure are related to genomic and non-genomic mechanisms. The pathological mechanisms induced by corticosteroids in the central nervous system (CNS) involve significant changes in neurotransmission, neurogenesis, and neuronal differentiation within key brain regions. These alterations can lead to various neuropsychiatric symptoms, including depression, anxiety, and cognitive impairments. Chronic corticosteroid exposure disrupts neurotransmitter balance, reduces neurogenesis, and increases oxidative stress and inflammation, contributing to neuronal damage and behavioral changes. The impact on hippocampal function is particularly notable, with evidence showing reduced hippocampal volume and impaired synaptic plasticity.

Translational science is mandatory for preventing neuropsychiatric symptoms following corticosteroid exposure. Given the pleiotropic actions of corticosteroids, finding a specific therapy can be challenging. So far, we reviewed several drugs that may reduce depression and psychosis-like behaviors, including agmatine, lithium, and antidepressants. The positive effects of these drugs are often attributed to their antioxidant properties. By providing the necessary substrates to buffer reactive species, these treatments might help prevent cell death. Implementing pretreatment before corticosteroid administration, such as using a widely available antioxidant drug like lithium, is worth considering. Additionally, for individuals with a known genetic predisposition to neuropsychiatric disorders, gene editing tools like CRISPR/Cas could be an option. Although gene expression therapies are relatively new, they have the potential to offer more specific and effective protection [74]. Understanding these mechanisms is crucial for identifying clinical manifestations and developing targeted interventions to mitigate the adverse effects of corticosteroids on mental health.

Table 1 summarizes the most important pathophysiological mechanisms involved in CIPDs based on the preclinical studies explained above.

## 3. Psychiatric Adverse Effects of Corticosteroids

### 3.1. History

Historically, CIPDs have been described in the literature since 1952 with reports of affective and psychotic manifestations [75]. However, the first large study that reported the association between psychiatric symptoms and corticosteroids was conducted by the Boston Collaborative Drug Surveillance Program in 1972 [76,77]. This study reported a correlation between the prednisone dose and CIPDs [76]. Since then, more cases have been reported, providing further evidence of the correlation between steroids and neuropsychiatric effects.

### 3.2. Epidemiology

CIPDs have been reported in all age groups [77,78]. The incidence of corticosteroid-induced psychiatric symptoms is very wide, ranging from 2% to 60% [6], and it is estimated to be seen in 5–18% of the patients treated with corticosteroids [79]. A recent systematic review and meta-analysis described the pooled proportion of CIPDs: 22% for depression, 11% for mania, 8% for anxiety, 16% for delirium, and 52% for behavioral changes [80]. Other symptoms, including violence, dangerous behaviors, self-injury, suicide ideation, and panic attacks, have been reported with low frequency in pediatric, adolescent, and young adult patients undergoing induction or consolidation therapy for hematologic malignancy [81]. Some studies have shown that the incidence of CIPDs appears to be more frequent in women than in men [82]; however, a recent systematic review by De Bock and Sienaert reports a lack of consensus to determine the independent risk factor based on gender. Further research is needed to thoroughly evaluate this lack of consensus [83]. A study conducted in the U.K. with adults registered between 1990 and 2008 at U.K. general practices contributing to the Health Improvement Network’s (THIN) primary care database found that, compared to patients with the same medical disease who were not treated with glucocorticoids, the hazard ratio for psychiatric symptoms was higher in subjects receiving glucocorticoids [84]. In fact, the hazard ratio for suicide or suicide attempt in patients with glucocorticoids was 6.89 (95% CI = 4.52–10.50); for depression, 1.83 (95% CI = 1.72–1.94); for mania, 4.35 (95% CI = 3.67–5.16); for delirium, confusion, or disorientation, 5.14 (95% CI = 4.54–5.82); and for panic disorder, 1.45 (95% CI = 1.15–1.85) [84].

### 3.3. Risk Factors Related to Patients

#### 3.3.1. Age and Gender

Studies in the current literature do not provide enough evidence to determine whether there is a higher risk of experiencing CIPDs based on gender [10,83,85,86]. Further research is needed to thoroughly evaluate this lack of consensus [83]. However, some evidence suggests that women are more susceptible to developing depression, in contrast to men, who are more susceptible to developing mania, confusion, or disorientation [9,84]. Regarding age, the risk of depression, mania, delirium, confusion, and disorientation increases with age, but suicidal behavior and panic disorder are more common in younger people [84]. However, the age range in relation to suicide risk is wide; in fact, there are also reports of patients with a risk of suicidal behavior between 18 and 50 years of age [9]. It was particularly identified that age could be an independent risk factor for severe CIPDs; the risk of CIPDs decreased with age [81]. Overall, a younger age (0–6 years old) seems to be a risk factor for behavioral problems, and an older age seems more of a risk factor for sleep problems [87].

#### 3.3.2. Medical History

Medical history, in particular associated comorbidities, is known to impact the individualized risk for CIPDs. Clinicians should inquire about overall psychiatric history; otherwise, it is suggested that psychiatric history or a history of steroid-related adverse psychiatric events could predict recurrence [9,83]. Other factors that may increase susceptibility to steroid-induced mania include a family history of bipolar disorder or psychosis and long-term treatment with steroids [83]. Systemic lupus erythematosus (SLE) has been associated with a higher risk of developing neuropsychiatric manifestations with corticosteroids compared to other autoimmune diseases [88]. In multiple studies, it was evidenced that SLE doubled the risk of mental alterations during corticosteroid management, mainly in high-dosage usage scenarios [83,89]. Fujieda and colleagues reported that anti-DNA/NR2 antibodies might be a predictive factor for post-steroid neuropsychiatric symptoms in patients with systemic lupus erythematosus [90]. Moreover, a possible association between blood–brain barrier (BBB) damage and CIPDs in patients with SLE may exist. Therefore, patients’ ongoing inflammatory processes, multiple systemic diseases, and brain lesions that affect BBB permeability might make them more susceptible to CIPDs [91]. More studies should be conducted to clarify the role of BBB dysfunction in CIPDs [92]. Patients with hematological malignancy may be at higher risk of developing CIPDs; chemotherapy agents, including intrathecal methotrexate and cranial irradiation, might increase BBB permeability. Moreover, chemotherapy agents may have a direct synergistic effect with steroids, affecting neuronal cell damage, particularly in the hippocampal area [93]. Lower creatinine, lower serum complement levels, and hypoalbuminemia have been reported to be associated with CIPD risk [94,95]. Other factors besides the use of steroids that may be related to the occurrence of psychiatric events, especially in the oncological population, include cancer itself, stress related to hospitalization, medical procedures such as lumbar puncture and central venous catheter placement, and the psychotic effects of anticancer agents [81].

#### 3.3.3. Drug Interactions

Drug interactions are also a risk factor for the development of CIPDs, above all cytochrome P450 (CYP3A) inhibitors [81] that can alter the metabolism of 6 beta-hydroxyprednisolone (a biologically active metabolite of prednisone), leading to increased corticosteroid levels and heightened development of psychiatric symptoms [95,96,97]. In fact, patients receiving a combination of corticosteroids and clarithromycin (a CYP3A4 inhibitor) have a higher risk of psychiatric symptoms. Higher age and polypharmacy can impact CYP3A4 activity due to changes in renal and liver metabolism, leading to altered steroid plasma concentrations. These physiological changes necessitate careful management by physicians to avoid adverse drug reactions and ensure therapeutic efficacy in older patients [83]. Attention to dosage adjustments, drug interactions, and monitoring liver and renal function is essential in this population.

#### 3.3.4. Genetics

Variations in genetics could explain some inter-individual variability in the glucocorticoid response and steroid-related toxicity [91]. In fact, single-nucleotide polymorphisms (SNP) related to the glucocorticoid receptor (GR) have been described in patients with CIPDs, and a positive association has been found between some polymorphisms of the *BCL-1* gene and the occurrence of affective symptoms during corticosteroid treatment [87]. Understanding these genetic influences is crucial for personalized medicine and tailoring treatments to minimize risks [74].

### 3.4. Risk Factors Related to Drug

#### 3.4.1. Route of Administration

CIPDs can develop independently of the route of administration of steroids (intra-nasal, topical, epidural, or intra-articular administration) [91]. It is unclear if the clinical presentation and severity of CIPDs are related to the route of administration [83], but some evidence suggests that there may be a higher risk of neuropsychiatric effects when the medication is administered intravenously (IV) compared to orally [94]. In fact, there have been cases where symptoms resolved when switching from IV to oral administration. Additionally, although the evidence is contradictory, Ogyu et al. found that patients who received IV methylprednisolone had a higher risk of developing neuropsychiatric effects [77]. No certain conclusions have been made about the route of administration as a risk factor for CIPDs, but one can assume based on the cases reported previously that systemic routes (mainly IV) should be used cautiously, considering the possibility of a major susceptibility to CIPDs [94]. Mood disorders seem to be more frequent with the use of systemic steroids, unlike cognitive disorders, which occur more frequently in nasal, inhaled, or dermal formulations [74,98].

#### 3.4.2. Dose

A high dose of corticosteroids is an important risk factor for developing CIPDs [83,99]. In the Boston Collaborative Drug Study performed in 1972, adults without a psychiatric history were examined, revealing an incidence of CIPDs at 1.3% with low to moderate doses <40 mg/day of prednisone or its equivalent, 4.6% with high doses of 41–80 mg/day, and escalating to 18.4% in patients receiving doses exceeding 80 mg/day [83]. Moreover, the average dose for developing psychosis is approximately 60 mg/day [95]. Currently, it is considered that a dose exceeding 30–40 mg/day or 1 mg/kg/day of prednisone or its equivalent represents an increased risk for the onset of CIPDs [97]. However, the dose range at which these symptoms can occur is very broad. In fact, cases of insomnia, hypomania, and elevated mood have been reported with doses as low as 2.5 mg per day [100]. The occurrence of specific CIPDs has been described as dose-dependent in some cases; panic disorders and delirium are more likely with daily prednisone doses of 11 mg, mania with 21 mg, depression with 40 mg, and psychotic symptoms with doses as low as 2.5 mg [100,101].

#### 3.4.3. Type of Corticosteroid

**Figure 2:**
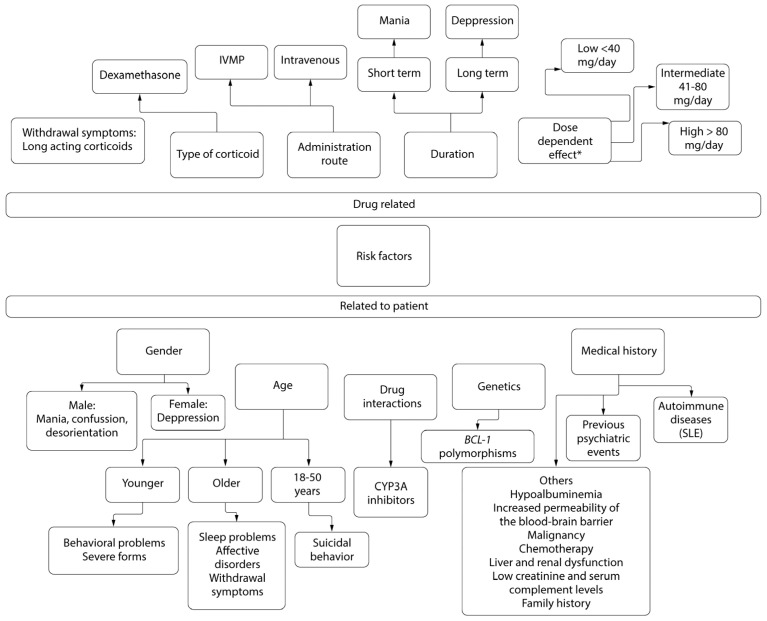
Risk factor for corticosteroid induced neuropsychiatric disorders. * Prednisone equivalents. Abbreviations: Intravenous methylprednisolone (IVMP), systemic lupus erythematosus (SLE).

Studies have documented that patients taking prednisolone have a higher risk of developing CIPDs [83]. However, it is important to note that this type of steroid is the most prescribed. Therefore, this is not sufficient to conclude that the use of prednisolone alone is a risk factor [83,102]. In their systematic review, De Bock and Sienaert found that prednisone, prednisolone, and methylprednisolone were most often associated with causing mania [83]. Some evidence suggests that patients receiving intravenous methylprednisolone (IVMP) have a higher incidence rate of CIPDs, which is significantly higher than patients receiving any other corticosteroid treatment [77]. Staub et al. researched CIPDs in pediatric, adolescent, and young adult patients undergoing induction or consolidation therapy for hematologic malignancies. They found that dexamethasone (DEX) use had a significantly higher incidence of CIPDs compared to prednisolone (PSL), accounting for 77.5% and 64.9%, respectively [81]. However, the high incidence of CIPDs reported in this study may be explained by the fact that the use of steroids as consolidation or induction therapy for hematologic malignancies requires higher doses compared to other diseases. This again suggests that higher doses may be associated with greater neuropsychiatric effects [81]. Another possible explanation for the higher incidence of CIPDs in patients using DEX is the greater penetration of dexamethasone into the cerebrospinal fluid (CSF) and its longer half-life in the CSF compared to other corticosteroids [103]. Figure 2 presents a summary of the risk factors for CIPDs, including both medication-related and patient-related factors, as well as the clinical presentation most frequently reported in the literature.

### 3.5. Clinical Presentation

The array of CIPDs is wide-ranging, covering affective and behavioral disruptions, psychotic episodes, cognitive deficiencies, and sleep problems. The most frequent conditions are depression (28–41%) and mania (29–35%), followed by psychosis (11–14%), delirium (10–13%), and mixed states (8–12%) [97]. Reversible dementia induced by corticosteroids has also been described, as well as a wide variety of symptoms such as dysphoria, withdrawal symptoms, obsessive compulsive spectrum symptoms, hetero aggressiveness, catatonia, panic attacks, and agoraphobia [74,97,104]. Mania is the most common symptom, characterized by distractibility, excitation, euphoria, hyperactivity, euphoria, and irritability [82,97]. Moreover, psychotic episodes can emerge in about 30–40% of cases during a manic episode [82,95]. The most common psychotic symptoms include auditory hallucinations, perplexity, disorganized behavior, confusion, delusions, or thought impairment. Furthermore, psychotic episodes have been found to occur more frequently in corticosteroid-related syndromes than in primary affective disorders [101]. It is essential to conduct follow-up even after resolving the psychotic episode, as steroid use has been observed to trigger schizophrenia in up to 3% of patients [74].

Cases of delirium and mild cognitive impairment have been described, manifesting as concentration difficulties, declarative memory alteration, and executive function impairment [47,91,95,97,98,105,106]. Regarding the duration of corticosteroid exposure, depressive symptoms and cognitive impairment are more common with chronic use, whereas acute use is associated with a higher frequency of manic symptoms [6,7,107,108,109]. Symptoms typically emerge within the first six weeks of treatment in up to 90% of patients, although they can develop within 3–5 days of initiation [82,97], with 60–85% occurring within the first week [95]. Complete improvement is anticipated in the great majority of patients following steroid withdrawal. Most will get better in two to six weeks, while some can take up to six months [95]. Additionally, the resolution time of symptoms may vary depending on the presentation of the episode, with psychotic symptoms and delirium having a shorter resolution time [10] compared to manic and depressive presentations, which typically take 2–3 weeks [110].

Regarding the differential diagnosis, the immediate onset of pruritus in the genital region reported by some patients is noteworthy. This phenomenon has been associated with phosphate ester in cases involving dexamethasone. Due to its implications, this adverse effect is often underreported by patients and has the potential to be misinterpreted by treating physicians as a psychiatric symptom [111].

In addition, the additive effect of corticosteroids in autoimmune diseases like lupus, multiple sclerosis, and others presents a significant challenge in distinguishing whether psychosis is a direct result of the autoimmune condition or an adverse effect of corticosteroid therapy. Corticosteroids, while effective in reducing inflammation and managing autoimmune symptoms, can induce psychiatric symptoms including psychosis. Therefore, careful clinical evaluation is essential to determine the underlying cause of psychiatric manifestations in patients undergoing corticosteroid treatment for autoimmune diseases. This differentiation is crucial for appropriate management and treatment strategies [112].

### 3.6. Diagnosis

Although there is no standardized classification or a specific diagnostic tool for CIPDs, it might be possible to classify these symptoms by assessing symptomatology utilizing the DSM-5 criteria considering psychiatric manifestations such as substance/medication-induced mental disorders [79,113,114,115,116]. Additionally, depending on the psychiatric symptom, it is possible to use other diagnostic tools that are frequently used to evaluate these symptoms in general but were not created specifically for the evaluation of CIPDs. The most frequently used scales are as follows: for depression, the Hamilton Rating Scale for Depression (HRSD) and Beck Depression Inventory (BDI); for mania, the Young Mania Rating Scale and the Activation Subscale of the Internal State Scale (AS-ISS); for anxiety, the Hospital Anxiety and Depression Scale (HADS) and Spielberger State Anxiety Index; for delirium, the Confusion Assessment Method (CAM); for behavioral changes in children, the Child Behavior Checklist (CBCL); and for psychosis, the Brief Psychiatric Rating Scale (BPRS) [80]. In the literature, up to 39 different scales have been recorded as being used in the context of CIPDs [80]. This reflects the need to create a tool that encompasses all clinical manifestations and can be useful in the context of CIPDs [80]. CIPD severity has also been evaluated with the Clinical Global Impressions-Severity of Illness Scale (CGI-S) [81].

The Naranjo score, developed in 1981, is a 10-item scale with good reliability and validity for predicting the probability of adverse reactions to drugs [117,118]. It was systematically developed to examine the causal relationship between a drug and an observed side effect. Each question has three possible answers (“yes”, “no”, or “do not know”), with a specific score determined, ranging from −1 to +2 depending on the item evaluated. It employs an interpretation system based on the final score, where a final result of 9 or more points indicates definite causality, 5–8 are determined to be probable causes, 1–4 are defined as possible, and 0 or less are considered doubtful [118]. It has been employed in diverse report cases regarding CIPDs but has not been widely validated.

Other scales assessing the causal relation between a drug and a suspected adverse effect have been proposed by the World Health Organization Collaborating Centre for International Drug Monitoring, the Uppsala Monitoring Centre [119]. Even though there is no standardized tool for CIPDs, no other scoring systems have been established otherwise, making the Naranjo score or the WHO scale a useful method to determine causality in patients with CIPDs.

### 3.7. Treatment

There is no specific or clear solution for managing patients with CIPDs; therapeutic measures should be individualized according to the severity of the CIPDs and the indication for corticosteroid therapy [94].

Below are some useful strategies to relieve symptoms [94]:

- Divide the dose into multiple doses per day.
- Decrease the dose.
- Switch from the IV to the oral route.
- Minimize the duration of therapy whenever possible. If the patient is receiving dexamethasone, it is suggested to switch to a lower-potency steroid. As mentioned earlier, dexamethasone probably has the highest penetration into the cerebrospinal fluid [94].

If the patient does not respond to steroid reduction or it is not possible to reduce the dose due to the patient’s condition, symptomatic treatment should be considered according to the patient’s clinical presentation [83].

#### 3.7.1. Specific Psychiatric Symptoms Management

● Mania Management:
  ○ The pharmacological treatment of CIPD is primarily supported by case reports and small studies, as no FDA-approved medications are available for managing these symptoms [120]. When extrapolating to the management of mania, mood stabilizers such as valproic acid, lithium, lamotrigine, and antipsychotics are recommended [120]. There is insufficient evidence to demonstrate the superiority of one medication over another, so the choice should be guided by the patient’s comorbidities and potential drug interactions. Among antipsychotics, atypical agents are preferred, with risperidone and olanzapine being first-line options [83]. It is important to note the adverse effects of lithium, including tremors, gastrointestinal disturbances, thyroid dysfunction, and weight gain [121]. Moreover, due to its narrow therapeutic index, lithium should be avoided in patients with renal disease, initiated at low doses, and serum lithium levels should be closely monitored [122].
● Depressive Symptoms Management:
  ○ Selective serotonin reuptake inhibitors (SSRIs) and serotonin-norepinephrine reuptake inhibitors (SNRIs) such as sertraline, fluoxetine, venlafaxine, and low-dose fluvoxamine are recommended [9]. SSRIs, such as sertraline and fluvoxamine, have the sigma-agonism effect, which enhances serotonin (5-HT) neurotransmission and contributes to its antidepressant effects. Studies have demonstrated that sigma-1 receptors can amplify 5-HT neurotransmission, exerting antidepressant effects [123]. It is recommended to avoid the use of tricyclic antidepressants (TCAs) since their anticholinergic effects may exacerbate delirium states [95,108], as well as increase agitation and psychosis [47].
● Psychosis Management:
  ○ Antipsychotics [9]. Second-generation antipsychotics such as risperidone and olanzapine are described as first-line treatments [83]. Antipsychotics with high anticholinergic activity should be avoided since they can worsen manic symptoms. Lithium is an option for managing depressive and manic states, but it should be avoided in patients with nephrotic syndrome. Special caution is necessary for patients with lupus or those who have undergone renal transplants [83].
● Cognitive impairment:
  ○ Cognitive impairment induced by steroids is hypothesized to be driven by heightened glutamatergic activity, leading to the exploration of drugs that decrease glutamatergic activity for potential therapeutic interventions [124]. Lamotrigine, a modulator of voltage-gated calcium and sodium channels, mitigates excitatory neurotransmitter release, particularly glutamate [124]. In a double-blind, placebo-controlled trial lamotrigine leads to better outcomes in relation to declarative memory in patients chronically exposed to corticosteroids [124]. This positive effect in cognitive performance can be explained by the anti-glutamatergic effect of lamotrigine, and the increased hippocampal dendritic outgrowth demonstrated in cell cultures induced by lamotrigine [124]. Similarly, memantine has shown potential efficacy in reversing declarative memory impairments induced by steroids, as demonstrated by improvements on the Hopkins Verbal Learning Test after treatment [47]. These cognitive enhancements are hypothesized to be due to memantine’s NMDA receptor antagonism, which modulates glutamatergic transmission, a key mechanism implicated in corticosteroid-related neurocognitive dysfunction [125]. Both drugs can be used safely when following standard pharmacological guidelines, considering their adverse effects and contraindications.
  ○ Acetylcholine levels are notably diminished in certain neuropsychiatric disorders, such as Alzheimer’s disease and delirium, which supports the use of cholinesterase inhibitors, like donepezil, to enhance cholinergic function [126]. While these drugs have demonstrated efficacy in chronic conditions, there is no substantial evidence supporting their use in acute settings [126]. Further research is warranted to explore the use of cholinesterase inhibitors in cognitive impairment induced by steroids.

**Figure 3:**
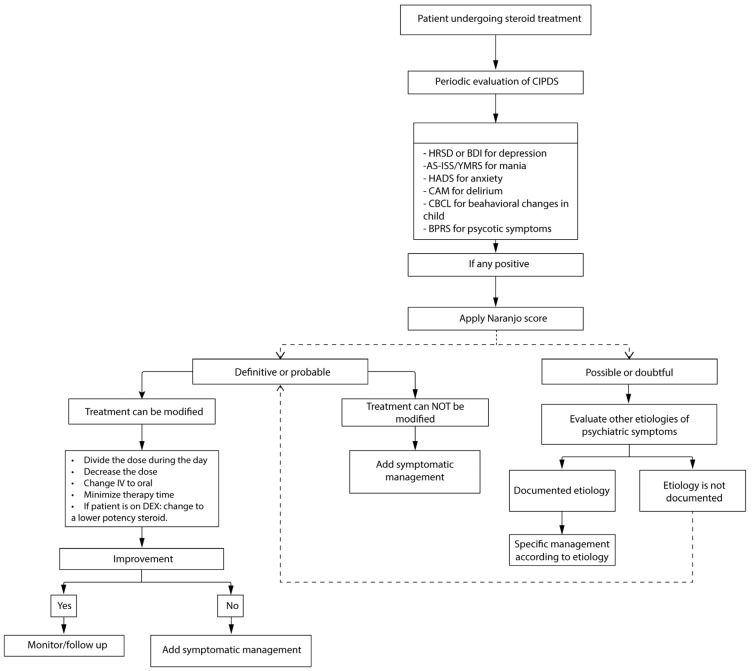
Treatment algorithm for corticosteroid-induced neuropsychiatric disorders. * For short-term therapy < 8 days: daily screening. For long-term therapy > 8 days: at least one weekly screening during the first 6 weeks and then monthly. Abbreviations: Corticosteroid-induced psychiatric disorders (CIPDs), Hamilton Rating Scale for Depression/Beck Depression Inventory (HRSD/BDI), Activation Subscale of the Internal State Scale/Young Mania Rating scale (AS-ISS/YMRS), Hospital Anxiety and Depression Scale (HADS), Confusion Assessment Method (CAM), Child Behavior Checklist (CBCL), Brief Psychiatric Rating Scale (BPRS).

In Figure 3, we present a diagnostic and management algorithm for patients with suspected CIPDs.

#### 3.7.2. Prevention

When initiating steroid therapy, it is crucial for medical staff to inform patients and their caregivers about the potential risk of CIPDs and to explain the symptoms that may appear during treatment to enable timely action [127]. Some interventions can reduce the likelihood of CIPDs, such as finding a balance between the safest and most effective dose based on the patient’s age, medical condition, risk factors, concomitant medications, and personal or family psychiatric history. It is advisable to avoid administering steroids close to bedtime to prevent insomnia [94]. For patients with risk factors or a previous history of CIPDs, the preventive use of olanzapine, lamotrigine or lithium has been described as useful. However, there are no large-scale controlled studies providing sufficient evidence to recommend these interventions [47,101,127].

Table 2 summarizes the most important CIPDs, including the medications most frequently involved, their relationship with dose, duration, and route of administration, as well as the usual clinical presentation of patients with these adverse events.

## 4. Discussion

The neuropsychiatric adverse effects associated with corticosteroids represent a pivotal intersection of neuroscience and clinical practice, underscoring the necessity for a comprehensive understanding among both neuroscientists and clinicians. The evidence synthesized in this review highlights the complex and multifaceted mechanisms through which corticosteroids influence the central nervous system (CNS), ultimately leading to a diverse spectrum of psychiatric manifestations.

At the cellular and molecular levels, corticosteroids significantly alter neurotransmission, neurogenesis, and neuronal differentiation, particularly within the hippocampus, cortex, and corticostriatal circuits. These alterations are evidenced by the modulation of key neurotransmitter systems, including serotonin, norepinephrine, and dopamine, which underpin the biochemical pathways involved in CIPDs. Importantly, these changes are not merely transient; chronic exposure to corticosteroids induces sustained modifications in gene expression and protein synthesis, which profoundly impact brain structure and function.

The hippocampus, a critical region implicated in cognitive processes, demonstrates notable vulnerability to corticosteroid-induced neurotoxicity. Chronic corticosteroid administration is associated with reductions in hippocampal volume, increased neuronal apoptosis, and dysregulation of the mammalian target of rapamycin (mTOR) signaling pathway, all of which contribute to the cognitive impairments observed in affected patients. The protective effects of agents such as agmatine and fluoxetine in mitigating hippocampal damage suggest potential therapeutic avenues for managing corticosteroid-induced neurotoxicity, emphasizing the need for further exploration in this area.

Clinically, the incidence of (CIPDs) varies considerably, influenced by factors such as dosage, duration of therapy, and individual patient susceptibility. The wide range of neuropsychiatric symptoms, encompassing affective disorders like depression and mania to more severe psychotic episodes, necessitates vigilant monitoring and proactive management by clinicians. The high prevalence of these symptoms, especially among patients receiving high doses or prolonged corticosteroid therapy, underscores the imperative for structured strategies aimed at risk mitigation. Strategies such as dose reduction, alternative routes of administration, or the use of lower-potency steroids like dexamethasone, when clinically appropriate, can significantly decrease the incidence of CIPDs. Additionally, identifying risk factors—including age, gender, genetic predispositions, and comorbid conditions—is critical for the development of individualized treatment plans.

While discontinuation or reduction in corticosteroid therapy remains the cornerstone of managing CIPDs, clinical constraints often render this approach impractical. Consequently, the use of psychotropic medications, including lithium, antipsychotics, and selective serotonin reuptake inhibitors (SSRIs), emerges as a viable alternative for symptom control. Although the efficacy of these pharmacological interventions is supported by limited studies, the findings highlight the necessity for more extensive research to optimize therapeutic regimens.

Looking ahead, future research should prioritize the elucidation of the precise molecular mechanisms underlying CIPDs, as well as the development of targeted interventions. The potential of gene-editing technologies, such as CRISPR/Cas, to offer more specific and effective protection against genetic predispositions to CIPDs represents a promising frontier. Furthermore, large-scale, controlled studies are essential to validate the efficacy of preventive strategies and therapeutic interventions identified in preclinical models, with the goal of improving patient outcomes in clinical settings.

## 5. Conclusions

Corticosteroids, while indispensable in treating a variety of medical conditions, pose a significant risk for developing neuropsychiatric adverse effects. The complexity of these effects, driven by both genomic and non-genomic mechanisms, necessitates a thorough understanding among clinicians to ensure timely identification and management. This review underscores the critical need for an interdisciplinary approach, integrating insights from neuroscience and clinical practice, to mitigate the risks associated with corticosteroid therapy and improve patient outcomes. Continued research and innovation in therapeutic strategies will be paramount in addressing this multifaceted clinical challenge.

## Figures and Tables

**Table 1:** Pathophysiological mechanisms involved in CIPDs.

Pathophysiological Mechanism	Description
Neurotransmission and Neurogenesis: Changes in neurotransmitters and neurogenesis	Corticosteroids regulate the expression of enzymes and hormones that modulate neurotransmitter and hormonal systems. Chronic administration in preclinical models alters cognition, reduces serotonin and norepinephrine, and decreases neurogenesis [12,13,14,15,16,17,18,19].
Neurotransmission and Neurogenesis: Amygdala atrophy	Corticosteroid-induced atrophy in the amygdala affects emotional regulation and autonomic responses [20].
Effects on the Hippocampus: Impact of glucocorticoids on hippocampal function	Sustained corticosterone administration reduces hippocampal volume, driven by oxidative stress and mTOR signaling alterations. Agmatine and fluoxetine can prevent this reduction [21,22,23,24,25,26,27,28,29,31,32].
Effects on the Hippocampus: Neurogenesis and corticosterone	Different concentrations of glucocorticoids modulate neurogenesis in the dentate gyrus, with effects dependent on telomerase activity [31,32].
Inflammation and Oxidative Stress: Increase in inflammation	Corticosteroids elevate inflammatory cytokines such as IL-1β, IL-6, and TNF-α, while flavonoids like quercetin can mitigate inflammation [33,34,35,36,37,38].
Inflammation and Oxidative Stress: Synaptic plasticity and BDNF	Brain-Derived Neurotrophic Factor (BDNF) is crucial for synaptic plasticity. Corticosterone affects BDNF levels, and administration of specific compounds can restore its function [39,40,41,42,43,44,45].
Mechanisms of Mania and Psychosis: Induction of mania by corticosteroids	Acute corticosteroid use can induce mania, related to oxidative damage and increased inflammation. Lithium has been shown to reduce these effects [46,47,48,49,50,51,52].
Mechanisms of Mania and Psychosis: Psychosis and dopaminergic pathways	Early life stress and corticosteroid exposure can alter dopaminergic pathways, increasing the risk of psychosis [53,54,55,56,57,58,59,60,61,63,64,65,66].
Excitotoxicity: Mechanism of excitotoxicity	Corticosteroids may increase glutamate transmission, leading to excitotoxicity and cell death [67,68,69,70,71,72,73].

**Table 2:** Clinical features of usual CIPDs.

Corticosteroid Type	Dose	Effects
Prednisone/Prednisolone	<40 mg/day	Low to moderate risk of CIPDs; incidence 1.3% [83,99].
Prednisone/Prednisolone	41–80 mg/day	Increased risk of CIPDs; incidence 4.6% [83,99].
Prednisone/Prednisolone	>80 mg/day	High risk of CIPDs; incidence 18.4% [83,99].
Methylprednisolone (IV)	High doses (varies)	Higher risk of CIPDs compared to other corticosteroids [77,94].
Dexamethasone	Varies (used in cancer)	Higher incidence of CIPDs; 77.5% in specific studies [81].
Prednisone	Low doses (e.g., 2.5 mg/day)	Can induce insomnia, hypomania, and elevated mood [100].
Prednisone	11 mg/day	Panic disorders, delirium [100,101].
Prednisone	21 mg/day	Mania [100,101].
Prednisone	40 mg/day	Depression [100,101].
Prednisone/Prednisolone	>30–40 mg/day or 1 mg/kg/day	Increased risk of CIPDs [97].
Methylprednisolone	Route: IV vs. Oral	Higher risk of neuropsychiatric effects when administered IV [94].
Prednisone/Prednisolone	Long-term treatment	Increased risk of steroid-induced mania [83].
Dexamethasone	Short-term/acute use	Manic symptoms [6,7].
